# Hyperglycemia-activated 11β-hydroxysteroid dehydrogenase type 1 increases endoplasmic reticulum stress and skin barrier dysfunction

**DOI:** 10.1038/s41598-023-36294-y

**Published:** 2023-06-06

**Authors:** Young Bin Lee, Hyun Jee Hwang, Eunjung Kim, Sung Ha Lim, Choon Hee Chung, Eung Ho Choi

**Affiliations:** 1 Department of Dermatology, Yonsei University Wonju College of Medicine, 20 Ilsan-ro, Wonju, 26426 Republic of Korea; 2 Department of Endocrinology and Metabolism, Yonsei University Wonju College of Medicine, Wonju, Republic of Korea; 3 Research Institute of Metabolism and Inflammation, Yonsei University Wonju College of Medicine, Wonju, Republic of Korea

**Keywords:** Diseases, Endocrinology, Pathogenesis

## Abstract

The diabetes mellitus (DM) skin shows skin barrier dysfunction and skin lipid abnormality, similar to conditions induced by systemic or local glucocorticoid excess and aged skin. Inactive glucocorticoid (GC) is converted into active glucocorticoid by 11β-hydroxysteroid dehydrogenase type 1 (11β-HSD1). Hyperglycemia in DM and excessive GC are known to increase endoplasmic reticulum (ER) stress. We hypothesized that hyperglycemia affects systemic GC homeostasis and that the action of skin 11β-HSD1 and GC contributes to increased ER stress and barrier defects in DM. We compared 11β-HSD1, active GC, and ER stress between hyperglycemic and normoglycemic conditions in normal human keratinocytes and *db/db* mice. 11β-HSD1 and cortisol increased with time in keratinocyte culture under hyperglycemic conditions. 11β-HSD1 siRNA-transfected cells did not induce cortisol elevation in hyperglycemic condition. The production of 11β-HSD1 and cortisol was suppressed in cell culture treated with an ER stress-inhibitor. The 14-week-old *db/db* mice showed higher stratum corneum (SC) corticosterone, and skin 11β-HSD1 levels than 8-week-old *db/db* mice. Topical 11β-HSD1 inhibitor application in *db/db* mice decreased SC corticosterone levels and improved skin barrier function. Hyperglycemia in DM may affect systemic GC homeostasis, activate skin 11β-HSD1, and induce local GC excess, which increases ER stress and adversely affects skin barrier function.

## Introduction

The skin plays a vital role in maintaining homeostasis and protecting against stressful conditions through its complex interactions of mediators, thereby performing various neuroendocine functions^1–3^. In particular, the skin, which is responsible for extra-adrenal and extra-gonadal steroidogenesis, requires a precise balance of glucocorticosteroids (GCs) to maintain proper innate immunity and skin barrier function^4,5^.

Local GC excess derived from dyshomeostasis of steroidogenesis in the skin leads to decreased skin thickness, and decreased collagen density in the dermis. The subsequent weakening of skin barrier function and delayed wound healing are consistent with the characteristics of cutaneous adverse effects caused by long-term topical GC use and the aging skin of the elderly^6–8^. Thus, the aging skin and prolonged use of topical GCs are considered local GC excess conditions.

The role of 11β-hydroxysteroid dehydrogenase (11β-HSD) in GC homeostasis has been highlighted. 11β-HSD type 1 (11β-HSD1) converts inactive GC to active GC, and 11β-HSD type 2 reverses this conversion^5^. The 11β-HSD is located at the membrane of the endoplasmic reticulum (ER) and is strongly expressed in the suprabasal layer of the epidermis^9^. The 11β-HSD expression in the skin (especially keratinocytes) is regulated by various stimuli, such as aging and UV exposure^9,10^. The 11β-HSD1 increase in the skin promotes the local steroidogenic pathway and inhibits the proliferation of keratinocytes^11,12^.

Patients with diabetes mellitus (DM) have a higher prevalence of xerosis and delayed wound healing due to compromised skin barrier function compared to the general population^13,14^. In our previous studies, we described the skin of patients with DM as an acceleration of aging due to impaired skin barrier function in patients with DM, similar to that of aging skin^13,15^.

ER stress activates an unfolded protein response to exert protective effects on normal ER function triggered by various extrinsic and intrinsic factors, including UV irradiation and oxidative stress^16,17^. In particular, the relationship between ER stress and advanced glycation end-product (AGE) and hyperglycemia has been reported^18,19^. Severe or prolonged ER stress leads to apoptosis signaling beyond cellular dysfunction, and C/EBP is known to be one of the factors mediating this pathway^20,21^.

However, the exact mechanism by which the increase in serum AGE induced by long-standing hyperglycemic conditions suppresses the proliferation of epidermal keratinocytes and eventually deteriorates skin barrier function remains unclear.

Therefore, as we hypothesized that the presence of DM would affect GC homeostasis, we investigated whether hyperglycemic conditions induce the dysregulation of the hypothalamus–pituitary–adrenal (HPA) axis and further elevation of cortisol. In addition, considering the role of 11β-HSD1 on keratinocytes, we aimed to determine the impact of 11β-HSD1 in local GC excess under in vitro and in vivo hyperglycemic conditions.

## Results

### Hyperglycemic condition induced the elevation of levels of 11β-HSD1, cortisol, and mRNA of RAGE, and the ER stress

**Figure 1:**
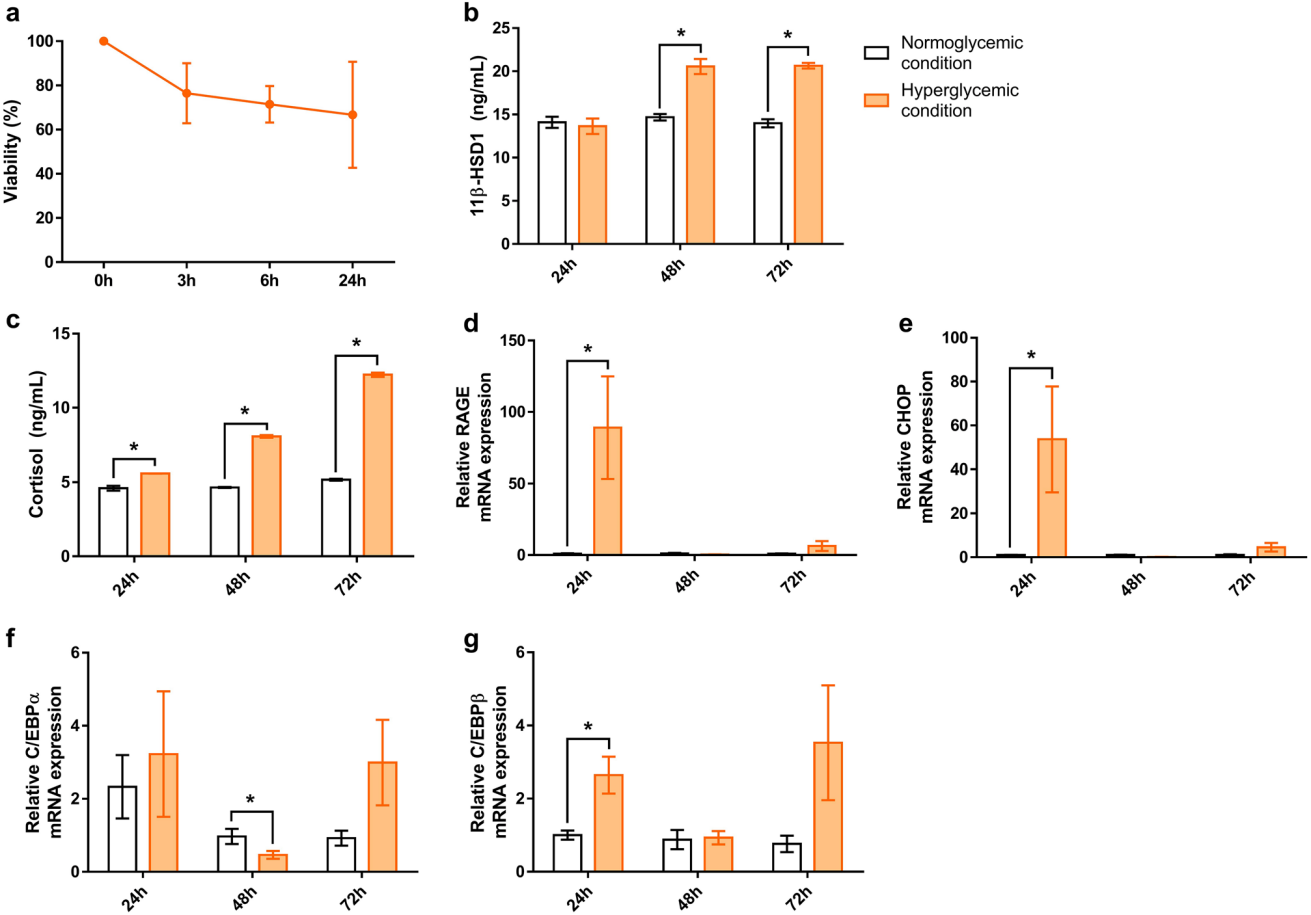
Hyperglycemic condition induced the elevation of the levels of 11β-HSD1, cortisol, and mRNA of RAGE, and the ER stress. Cell viability of NHK was evaluated up to 72 h at 24-h intervals after glucose treatment (**a**). 11β-HSD1 levels in NHK under hyperglycemic conditions (treated with 26 mmol/L of D-glucose) were significantly higher than those in normoglycemic conditions (treated with 6 mmol/L of D-glucose) (**b**). A time-dependent increase in cortisol was observed in the hyperglycemic condition; no increase was observed in the normoglycemic condition (**c**). The RAGE and CHOP mRNA expression significantly increased at 24 h and decreased from 48 h in the hyperglycemic condition; the change under normoglycemic conditions was insignificant (**d**, **e**). The C/EBP mRNA expressions were high at 24 h, decreased at 48 h, and increased at 72 h in hyperglycemic conditions; these patterns were unapparent under normoglycemic conditions (**f**, **g**). GAPDH was used as an internal control (**d**–**g**). Bars indicate the mean ± SE (N = 3; *p < 0.05, Student’s *t*-test).

Hyperglycemia resulted in a gradual decrease in cell viability over time. The viability was < 80% at 24 h in normal human epidermal keratinocytes (NHKs) under hyperglycemic conditions (Fig. 1a).

Protein and mRNA levels were compared between normoglycemic and hyperglycemic conditions in cell culture. The 11β-HSD1 and cortisol levels increased with time in NHKs under hyperglycemic conditions. In contrast, a quantitative increase in 11β-HSD1 and cortisol with time was not observed in NHKs under normoglycemic conditions. The difference in the amount between the normoglycemic and hyperglycemic conditions at each time point was significant (Fig. 1b,c).

The mRNA levels of epidermal receptor of AGE (RAGE) and CHOP (terminal marker of ER stress) showed a steep increase in the hyperglycemic condition in the first 24 h compared to the normoglycemic condition, which then decreased after 48 h. Under hyperglycemic conditions, a slight rebound was observed at 72 h. Significant changes in the hyperglycemic condition were not observed under normoglycemic conditions (Fig. 1d,e). The mRNA levels of C/EBPα and C/EBPβ showed a similar curve in the hyperglycemic condition. However, under normoglycemic conditions, the change with time was relatively weak (Fig. 1f,g).

### 11β-HSD1 knockdown suppressed the elevation of cortisol levels with a partial decrease of ER stress in the hyperglycemic condition

**Figure 2:**
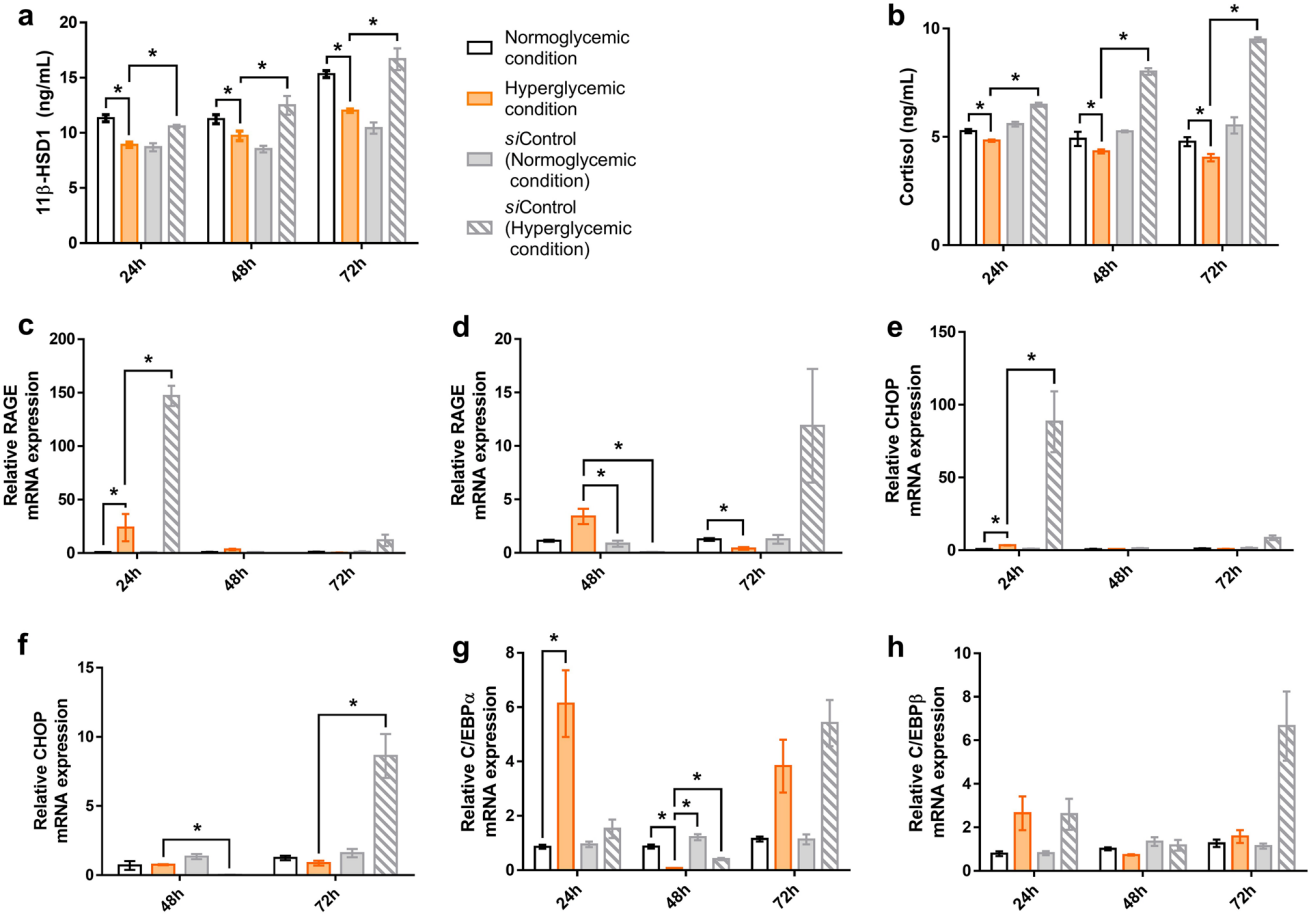
No increase in cortisol level and a partial decrease in ER stress were observed in NHKs transfected with 11β-HSD1 siRNA. NHK cultures were transfected with 11β-HSD1 siRNA, and 11β-HSD1, cortisol, RAGE, C/EBP, and CHOP levels were compared at 24-h intervals according to the glucose concentration for up to 72 h. Transfection was confirmed by reduction in the amount of 11β-HSD1 (**a**). Cortisol levels in hyperglycemic conditions were significantly lower than those in normoglycemic conditions at all time points (**b**). Significant relationships between hyperglycemic and normoglycemic conditions or *si*Control (hyperglycemic condition) are denoted by an asterisk, while other correlations are detailed in supplemental materials (**b**). The RAGE and CHOP mRNA expression increased rapidly at 24 h and decreased after 48 h in hyperglycemic conditions, but the change was weak under normoglycemic conditions (**c**, **e**). The relative mRNA expression levels of RAGE and CHOP at 48- and 72-h data were separately extracted and presented in additional graphs (**d**, **f**). The mRNA expression of C/EBP was high at 24 h, decreased at 48 h, and increased at 72 h in hyperglycemic conditions. The expression pattern was not observed under normoglycemic conditions (**g**, **h**). GAPDH was used as an internal control (**c**–**h**). Bars indicate the mean ± SE (N = 3; *p < 0.05, one-way ANOVA followed by the Bonferroni-Dunn test for multiple comparison). *si*Control; scrambled siRNA control.

The mRNA and protein levels were compared between hyperglycemic and normoglycemic conditions in the 11β-HSD1 siRNA-transfected NHKs. Following transfection, the amount of 11β-HSD1 decreased, and this decrease was more pronounced under hyperglycemic conditions than under normoglycemic conditions (Fig. 2a). In the siRNA-transfected cells, cortisol levels tended to decrease with time (Fig. 2b). Notably, this tendency was far different from the time-dependent increase of cortisol in non-transfected NHKs under hyperglycemic conditions (Fig. 1c). At all times, the amount of cortisol was significantly lower in the hyperglycemic condition than in the normoglycemic condition, indicating that the action of 11β-HSD1 is more pronounced in hyperglycemia than in normoglycemia.

In the siRNA-transfected cells, RAGE, CHOP, and C/EBP mRNA showed an increase at 24 h, the lowest at 48 h, and re-increase at 72 h, similar to the non-transfected condition (Fig. 2c–h). Although the expression pattern in the transfected condition was similar to that shown in the non-transfected condition, the absolute value of RAGE and CHOP mRNA expression in the hyperglycemic condition was relatively lower in the 11β-HSD1 siRNA-transfected condition than in the non-transfected or scrambled siRNA control (Fig. 2c–f). Therefore, the 11β-HSD1 knockdown in the hyperglycemic condition could suppress the elevation of cortisol levels with decrease in ER stress.

### Increase in ER stress under hyperglycemic conditions within the first 24 h was not significantly suppressed by 11β-HSD1 inhibitor treatment, but showed a tendency to alleviate the elevation

Before the experiment, we found that the 11β-HSD1 inhibitor itself could affect cell viability. Absolute toxicity was observed when it exceeded a certain concentration (Supplementary Fig. 1). To specifically evaluate the relationship of 11β-HSD1 with RAGE, CHOP, and C/EBP observed in the 11β-HSD knockdown condition, we treated NHKs under hyperglycemic conditions with 0.01 µM 11β-HSD1 inhibitor.

**Figure 3:**
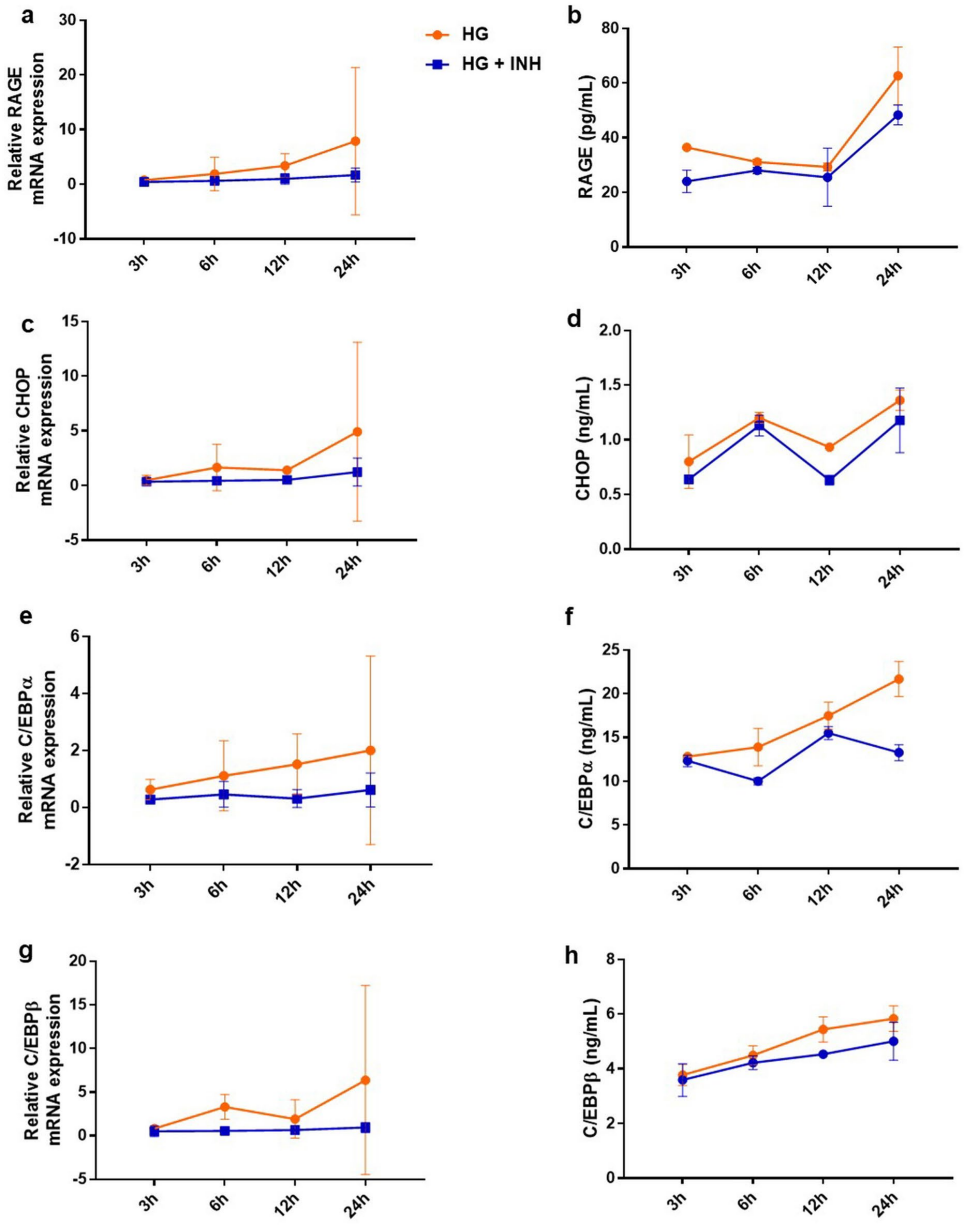
Increasing the expression of RAGE, CHOP, and C/EBP up to the first 24 h under the hyperglycemic condition was partially suppressed by the 11β-HSD1 inhibitor. Changes in mRNA expression and protein levels were observed at 3-h intervals up to 24 h after treatment with 11β-HSD1 inhibitor in NHKs under hyperglycemic conditions. The mRNA expressions and protein levels of RAGE and CHOP in hyperglycemic conditions showed a tendency to increase with time but did not show a statistically significant difference compared to the 11β-HSD1 inhibitor-treated condition (**a**–**d**). The mRNA expressions and protein levels of C/EBPα and C/EBPβ showed a gradual increase in hyperglycemic conditions over time, but these changes were insignificant in inhibitor-treated conditions (**e**–**h**). GAPDH was used as an internal control (**a**, **c**, **e**, **g**). The dots with error bars indicate the mean ± SE (N = 3). HG; hyperglycemia, HG + INH; hyperglycemic condition treated with an 11β-HSD1 inhibitor.

Although significant temporal changes in RAGE, CHOP, and C/EBP were inconsistently observed with or without 11β-HSD1 inhibitor treatment, the expression of the three tended to increase over time under hyperglycemic conditions within 24 h. Despite the absence of prominent differences between the treated and untreated NHKs, the increase in RAGE, CHOP, and C/EBP over time showed a tendency to be weakened in NHKs treated with 11β-HSD1 inhibitor (Fig. 3).

### Inhibition of ER stress effected down-regulation of 11β-HSD1 and cortisol under hyperglycemic condition

**Figure 4:**
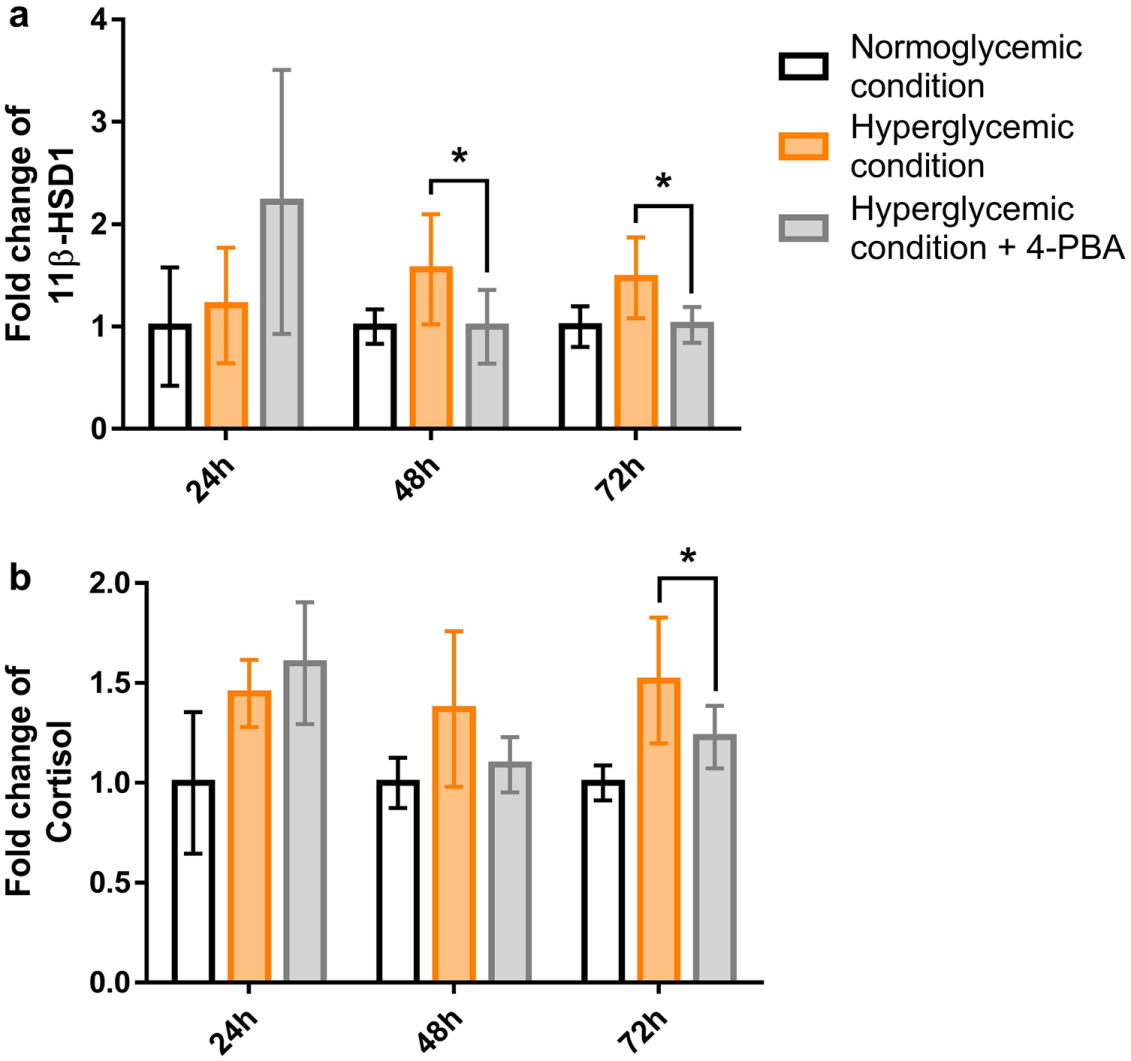
The inhibition of ER stress suppressed the secretion of 11β-HSD1 and cortisol stimulated by hyperglycemia. NHKs under hyperglycemic condition were treated with 4-Phenyl butyric acid (4-PBA), an ER stress inhibitor. When the value under normoglycemic conditions was 1, the change in each condition was expressed as a “fold change”. In NHK treated with 4-PBA, under hyperglycemic conditions, the level of 11β-HSD1 was significantly lower than that of the untreated NHKs under the same hyperglycemic condition after 48 h (**a**). After 72 h, the level of cortisol was also significantly lower in 4-PBA treated NHKs than in untreated NHKs (**b**). Significant relationships between hyperglycemic condition and hyperglycemic condition treated with 4-PBA are denoted by an asterisk. Further detailed *post-hoc* analysis results can be found in the supplemental materials. GAPDH was used as an internal control (**a**, **b**). Bars indicate the mean ± SE (N = 8; *p < 0.05, one-way ANOVA followed by the Bonferroni-Dunn test for multiple comparison).

To investigate the impact of ER stress on 11β-HSD1 expression, changes in cortisol and 11β-HSD1 levels in NHKs were examined after treatment with 2 µM 4-PBA, an ER stress inhibitor (Supplementary Fig. 2), under hyperglycemic conditions. Time-dependent increase patterns of both 11β-HSD1 and cortisol levels were not observed in NHKs when treated with 4-PBA under hyperglycemic conditions (Fig. 4a,b). In particular, 11β-HSD1 and cortisol levels of hyperglycemic condition treated with 4-PBA were significantly lower than those of untreated after 48 h.

In addition, we examined the changes after treatment with thapsigargin (TG), an ER stress activator, in NHKs under normoglycemic conditions (Supplementary Fig. 3). TG-treated NHKs showed increasing CHOP levels with increasing concentrations of TG. After treatment with TG, the concentration of 11β-HSD1 was also higher than that in the control. However, a quantitative increase in 11β-HSD1 expression according to the concentration of TG was not observed. Therefore, ER stress plays a pivotal role in inducing changes in 11β-HSD1 and cortisol.

### Hyperglycemic condition, high ER stress state, increased keratinocyte differentiation in short period, at least 72 h

**Figure 5:**
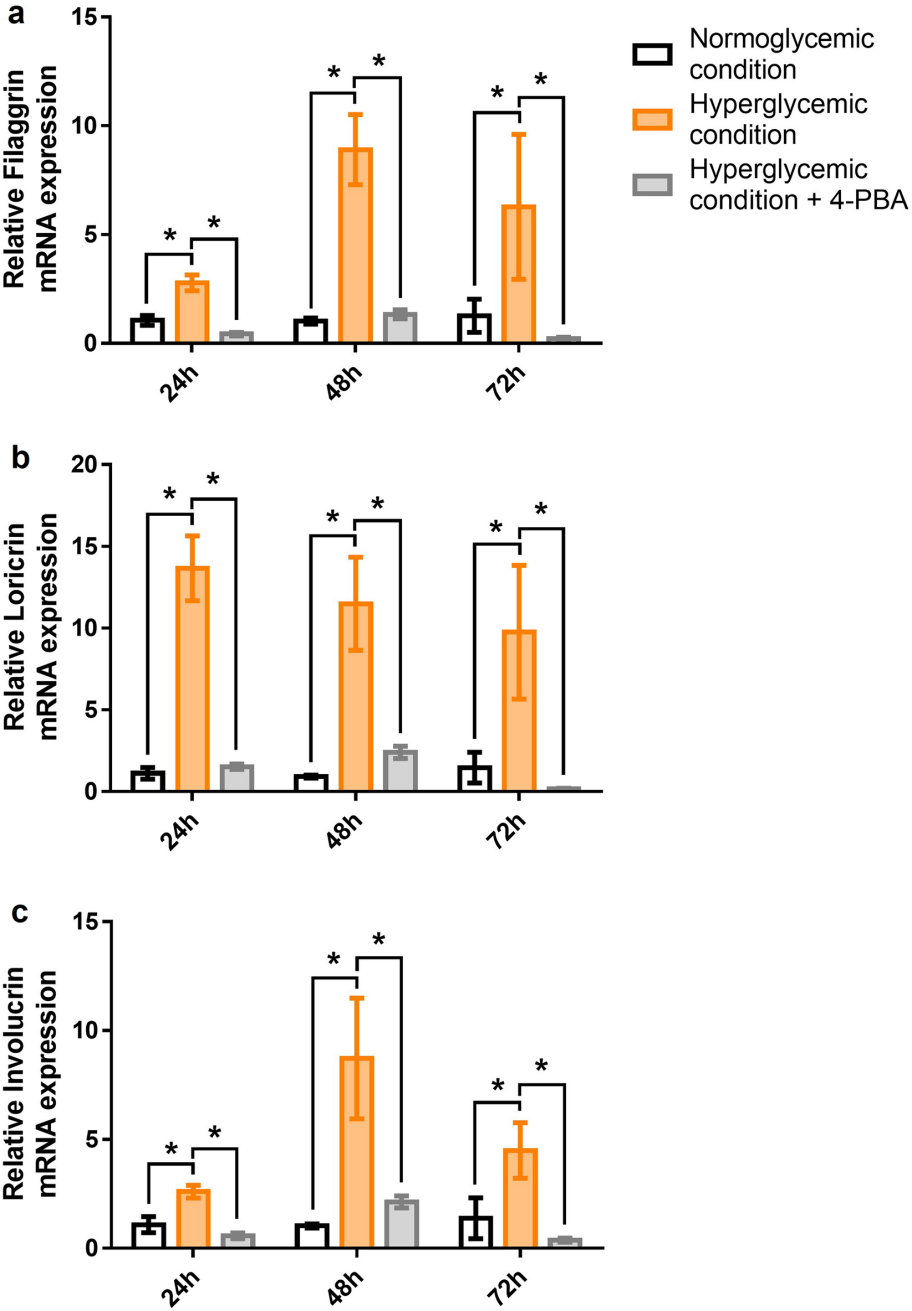
Hyperglycemia increased keratinocyte differentiation, the effect of which was reduced by inhibition of ER stress. The mRNA level of filaggrin in NHK under hyperglycemic condition was significantly higher than that under normoglycemic conditions at all time points (**a**). The mRNA levels of loricrin and involucrin were significantly higher than those observed in NHKs under normoglycemic condition (**b**, **c**). In NHKs under hyperglycemic condition treated with 4-PBA, all three parameters were significantly decreased compared to the untreated NHKs. GAPDH was used as an internal control (**a**–**c**). Bars indicate the mean ± SE (N = 5; *p < 0.05, Student’s *t*-test). 4-PBA; 4-Phenyl butyric acid.

Changes in keratinocyte differentiation markers including filaggrin, loricrin, and involucrin were evaluated in NHKs at different glucose concentrations. From 24 h onward, the mRNA levels of filaggrin, loricrin, and involucrin in NHKs under hyperglycemic condition were significantly higher than those in NHKs under normoglycemic condition (Fig. 5a–c). This elevation was maintained for up to 72 h. In NHKs under hyperglycemic condition treated with 4-PBA, all three marker levels were significantly lower than those under hyperglycemic condition without 4-PBA treatment.

### Fourteen-week-old *db/db* mice showed different hormonal profile compared to 8-week-old *db/db* mice

**Figure 6:**
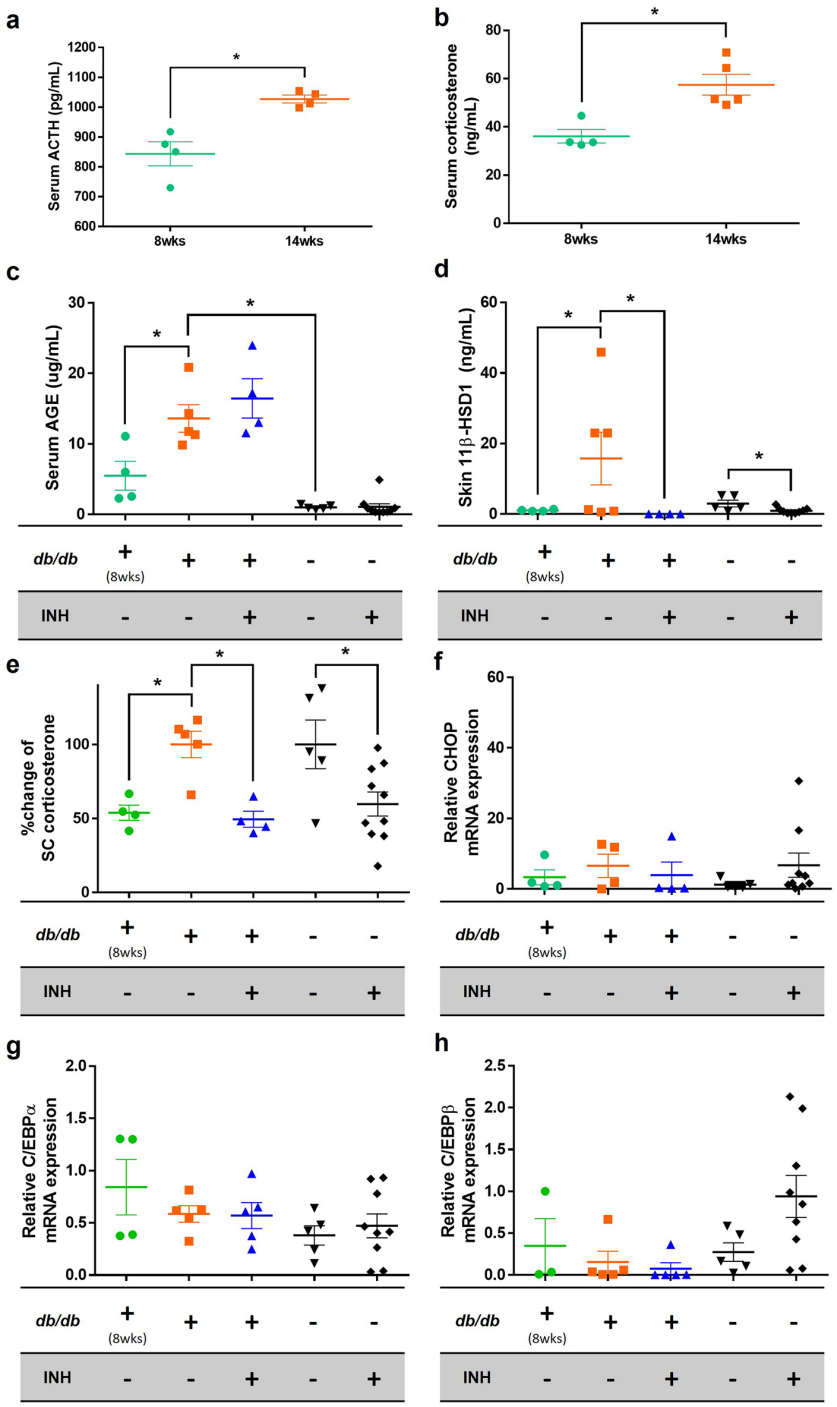
Serum levels of ACTH, corticosterone, and AGE and skin levels of 11β-HSD1 and corticosterone were increased in 14-week-old *db/db* mice compared to 8-week. Skin 11β-HSD1 and corticosterone production were inhibited by a topical 11β-HSD1 inhibitor. Serum ACTH and corticosterone levels were compared between 8-week-old and 14-week-old *db/db* mice (N = 5 per group; *p < 0.05, Student’s *t*-test) (**a**, **b**). Serum AGE, skin 11β-HSD1, SC corticosterone, skin CHOP mRNA expression, and skin C/EBP mRNA expression levels were compared 2 weeks after topical application of vehicle and 11β-HSD1 inhibitor in *db/db* mice and controls (**c**–**h**). When the average corticosterone level of mice untreated with topical 11β-HSD1 inhibitor within each strain was 100, the amount of change in each group was expressed as “% change of SC corticosterone” (**e**). GAPDH was used as an internal control (**f**–**h**). The scatter plot with error bars indicates the mean ± SE (N = 5 per group, except for the group with controls treated with 11β-HSD1 inhibitor (N = 10); *p < 0.05, one-way ANOVA followed by the Bonferroni-Dunn test for multiple comparison)*. db/db*; *db/db* mice. INH; 11β-HSD1 inhibitor treatment.

The 14-week-old *db/db* mice showed higher serum glucose level, weight, and serum AGE than the controls, suggesting that the setting of *db/db* mice with DM condition was appropriate (Supplementary Fig. 4). Serum corticosterone and ACTH levels in 14-week-old *db/db* mice were significantly higher than those in 8-week-old *db/db* mice (Fig. 6a,b). It seems that hyperglycemia induces the activation of the HPA axis.

### Elevated 11β-HSD1 and corticosterone were observed in 14-week-old *db/db* mice, similar to the in vitro results

**Figure 7:**
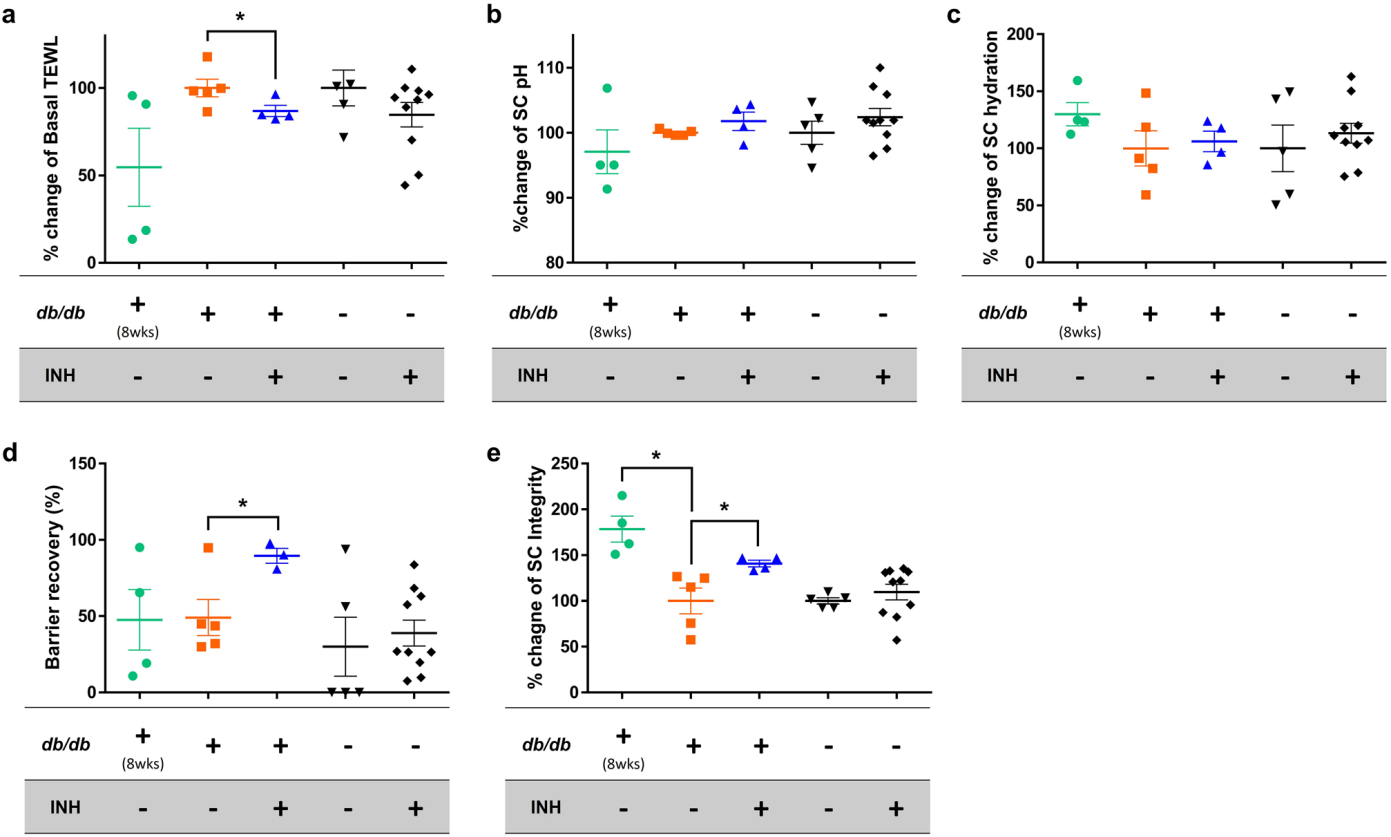
Skin barrier function can be improved by applying the topical 11β-HSD1 inhibitor in *db/db* mice. Skin barrier function was measured 2 weeks after topical application of the vehicle and 11β-HSD1 inhibitor in *db/db* mice and controls. Evaluation of skin barrier function included basal TEWL, SC pH, SC hydration, barrier recovery, and SC integrity (**a**–**e**). The 14-week-old *db/db* mice treated with topical 11β-HSD1 inhibitor showed a significantly lower TEWL, higher barrier recovery rate, and higher SC integrity compared to the 14-week-old *db/db* mice treated with the vehicle (**a**, **d**, **e**). When the average level of mice untreated with topical 11β-HSD1 inhibitor within each strain (*db/db* and control) was 100, the amount of change in each group was expressed as “% change”. The scatter plot with error bars indicates mean ± SE (N = 5 per group, except for the group with controls treated with 11β-HSD1 inhibitor (N = 10); *p < 0.05, one-way ANOVA followed by the Bonferroni-Dunn test for multiple comparison). *db/db*; *db/db* mice. INH; 11β-HSD1 inhibitor treatment.

Compared to 8-week-old *db/db* mice, 14-week-old *db/db* mice showed relatively higher serum AGE, skin 11β-HSD1, and stratum corneum (SC) corticosterone (Fig. 6c–e). However, there was no significant difference in CHOP and C/EBP levels between the two groups (Fig. 6f–h). In terms of skin barrier function, a significant decrease in SC integrity was observed in 14-week-old *db/db* mice compared to that in 8-week-old *db/db* mice (Fig. 7e). Basal transepidermal water loss (TEWL) and SC pH tended to increase in 14-week-old *db/db* mice (Fig. 7a,b). These results suggest that the long-standing condition of DM acts as a precursor to 11β-HSD1 and cortisol elevation, and consequently may induce abnormal skin barrier function at the mouse level.

### Inhibition of skin corticosterone production and improvement of skin barrier function were observed in 14-week-old *db/db* mice treated with a topical 11β-HSD1 inhibitor

After applying the vehicle and a topical 11β-HSD1 inhibitor for 2 weeks, protein and mRNA expressions in the skin and skin barrier function were measured. The level of SC corticosterone was lower in 14-week-old *db/db* mice treated with a topical 11β-HSD1 inhibitor than in 14-week-old *db/db* mice treated with the vehicle. Even in the control group, mice treated with the topical 11β-HSD1 inhibitor showed lower levels of SC corticosterone than vehicle-treated mice.

No significant difference in epidermal C/EBP was observed between the topical 11β-HSD1 inhibitor-treated and vehicle-treated 14-week-old *db/db* mice (Fig. 6g,h). Notably, the CHOP level of 14-week-old *db/db* mice treated with the 11β-HSD1 inhibitor showed a decreasing tendency, although not significant, compared to that of vehicle-treated mice (Fig. 6f).

The 14-week-old *db/db* mice treated with topical 11β-HSD1 inhibitor showed a lower basal TEWL, higher barrier recovery rate, and higher SC integrity than the vehicle-treated mice (Fig. 7a, d, e), although skin pH and SC hydration were not significantly different (Fig. 7b, c). Accordingly, it is suggested that inhibition of skin 11β-HSD1 significantly improves epidermal defects by alleviating local GC excess.

## Discussion

In vivo and in vitro studies have shown that hyperglycemic conditions cause structural and functional defects in the epidermis in both non-wounded and wounded skin^13,15,22,23^. However, these findings are mainly based on phenomenological observations, and the specific mechanisms regarding the causal relationship between hyperglycemia and impaired skin barrier function remain mostly unknown. Although various signaling pathways are presumed to be involved in the process by which high glucose conditions induce metabolic abnormalities in endocrinological fields, studies focusing on keratinocytes are limited.

HPA axis activation and elevation of systemic GC induced by hyperglycemic conditions were observed in this study. Although it is debatable whether the metabolic syndrome characterized by insulin resistance elevates systemic cortisol levels clinically, there is at least a consistency in the HPA axis abnormalities including aberrant cortisol response to external stress^24–27^. Thus, it is speculated that DM drives the systemic balance of GC in a steroidogenic (hypercortisolemia) manner.

The identification of key enzymes involved in steroidogenesis, which converts cholesterol into steroid hormones, and the understanding of the subsequent signaling pathways leading to GC production in the skin, akin to those observed in classic steroidogenic tissues like gonads and adrenal glands, support the notion of the skin’s physiological role and independent status as a peripheral neuroendocrine organ^2,3^. We previously reported that activating the HPA axis by systemic stimuli, such as psychological stress, could affect local GC activation^28^. Furthermore, it is thought that the skin has its own cycle corresponding to the systemic HPA axis or interactive crosstalk^29–33^. Accordingly, systemic GC excess in the background of DM may lead to a mainstream stimulation of local GC production (higher secretion of cortisol in keratinocytes).

The qualitative and quantitative increase in 11β-HSD activity, which enhances the local availability of GC, attenuates the acute inflammation of keratinocytes^34–36^. The regulation of 11β-HSD1 has been shown to be influenced by various environmental stimuli, contributing to the maintenance of epidermal barrier homeostasis. For instance, studies have demonstrated the activation of 11β-HSD1 in response to UV exposure in human skin ex-vivo^37^. Additionally, locally dysfunctional steroidogenic pathways have been clinically observed in chronic inflammatory dermatosis, such as atopic dermatitis and psoriasis^36,38^. It has been demonstrated in aged mice that chronically activated 11β-HSD1 plays an important role in age-related epidermal dysfunction and elevation of cortisol^7,39^. Considering that the epidermal alteration in DM corresponds to boosted aging skin^13,15^, 11β-HSD1 activation in the setting of DM shown in this study may superimpose the acceleration of local GC excess. Therefore, it can be assumed that 11β-HSD1 induced GC elevation disrupts GC homeostasis and leads to gradual GC excess in local steroidogenesis of DM, in addition to mainstream GC excess driven by HPA axis activation.

In contrast to the relatively well-established association between ER stress and hyperglycemia, studies on the relationship between ER stress and 11β-HSD1 in the skin are limited. Based on the consistent changes in 11β-HSD1 and cortisol levels in NHKs by regulation of ER stress in this study, hyperglycemia-triggered high ER stress mediates a mainstream process including 11β-HSD1-mediated cortisol production. Furthermore, an increased keratinocyte differentiation, induced by high ER stress, is in line with the findings of previous studies of pathological keratinization disorders with aberrant ER stress^40–42^.

However, aside from the regulation of 11β**-**HSD1 by ER stress, 11β-HSD1-inhibition suppressed ER stress to some extent in this study. This suggests that 11β-HSD1-mediated cortisol production is not a sub-signaling step unilaterally governed by ER stress. In terms of the possibility of a link between ER stress and GC (or 11β**-**HSD1), the following possibilities can be considered.

There are conflicting results regarding the relationship between GC and ER stress^43,44^. GC was also reported to increase ER stress under certain conditions showing tissue-specificity such as in trabecular meshwork cells in GC-induced glaucoma, in hippocampus with overexposure of endogenous GC during chronic stress, and in endotheliocytes with GC-induced osteonecrosis of femoral head^45–47^. GC was found to active certain signaling pathways, including the IRE1α/XBP-1ER and autophagy^48,49^. This suggests functional complexity between ER stress and the GC signaling pathway. Hence, it seems that inhibition of 11β-HSD1 weakened additional GC elevation and eventually led to a decrease in ER stress in the study.

Altogether, hyperglycemia in DM induces high ER stress and a systemic GC surge through HPA axis activation, which is the cornerstone of local GC elevation. An increase in ER stress drives 11β-HSD1-mediated GC production which exacerbates local GC excess. ER stress is bidirectionally influenced by the GC signaling pathway. Local GC excess including increased GC by 11β-HSD1 activation causes increased epidermal differentiation. However, prolonged GC excess leads to the suppression of keratinocyte proliferation which has a detrimental effect on the epidermis, resulting in skin barrier dysfunction^15,50^.

Nevertheless, several points in this study need to be interpreted carefully. First, the decrease in viability following the prolonged cell-breeding period can cause an increase in ER stress through a complex mechanism related to unintentional cell apoptosis. Consequently, accurate quantitative evaluation of the pure increment of ER stress due to hyperglycemia is limited. Regardless of the presence or absence of the skin HPA axis, among the production of systemic GC excess-driven local GC, it is difficult to accurately estimate the proportion of GC that 11β-HSD1 is responsible for. Conversely, there is a limit to quantifying the effect of local GC production on the systemic HPA axis via or non-via 11β-HSD1. However, because body weight, glucose, and serum AGE did not change in the murine study even after 2 weeks of topical 11β-HSD1 inhibitor application, it is quite clear that inhibition of skin 11β-HSD1 is a sufficient tool to control local GC excess without significant systemic changes.

In conclusion, DM could directly accelerate the impairment of skin barrier function with an increase in ER stress. Furthermore, DM is thought to induce systemic and local GC synergy, accompanied by higher local GC excess due to the action of 11β-HSD1. Thus, the clinical efficacy for the improvement of skin barrier dysfunction in DM through the inhibition of 11β-HSD1 is expected.

## Methods

### Tissue preparation

Normal human epidermal keratinocytes (NHK, 50,000 cells/mL) were cultured in EpiLife™ medium (Thermo Fisher Scientific, Waltham, MA, USA) supplemented with antibiotic–antimycotic and human keratinocyte growth supplement (Thermo Fisher Scientific) at 37 °C under 5% CO_2_. Third passage keratinocytes were utilized for the experiment. After 3 days of cell seeding, normoglycemic and hyperglycemic conditions were established by D-glucose treatment. As described in the literature, normoglycemic and hyperglycemic conditions were set for D-glucose at concentrations of 6 mmol/L and 26 mmol/L, respectively^51–53^. After 3 days of D-glucose treatment, the amounts of protein and mRNA were detected in the culture medium. The reagents used for intervention in cell culture were as follows; The selective 11β-HSD1 inhibitor, CAS 1009373-58-3 (Merck & Co., Kenilworth, NJ, USA) and 4-Phenyl butyric acid (4-PBA), P21005 (Merck & Co., Kenilworth, NJ, USA). All methods were carried out in accordance with relevant guidelines and regulations.

### Transfection with siRNA

A day prior to transfection, the cells were seeded on type-1 collagen-coated plates. The cells were transfected with 50 nM 11β-HSD1 siRNA or control siRNA (Bioneer, Daejeon, Korea) using a mixture of Opti-MEM and Lipofectamine RNAiMAX reagent (Invitrogen, Carlsbad, CA, USA). The culture medium was replaced 6 h later. Cells were then used for the experiments 48 h after transfection.

### Real-time reverse transcription PCR (RT-PCR) of mRNA

Total mRNA was isolated from NHKs using QuantiTect Reverse Transcription Kits (Qiagen, Hilden, Germany). The product was then reverse-transcribed into first-strand complementary DNA (cDNA). Approximately 60 ng of cDNA was used as the template for each reaction. The housekeeping gene *GAPDH* was used as an internal reference to normalize the data, accounting for any differences in sampling. All PCR reactions were performed in triplicate, and the results were expressed as the mean of values obtained from three separate experiments. Amplification of samples were carried out using the primers outlined in Table S1 under the following conditions: initial denaturation at 95 °C for 15 min, followed by 45 cycles of 95 °C for 15 s and annealing/extension 60 °C for 1 min. Further details can be found listed in Supplemental materials.

### Enzyme-linked immunosorbent assay (ELISA)

The culture supernatant was centrifuged for 15 min at 1000 × *g*, 2–8 °C. The amount of cortisol in the samples was measured using a Human Cortisol ELISA Kit (Cusabio, Houston, TX, USA) according to the manufacturer’s protocol. The expression of 11β-HSD1 was measured using the Human HSD11B1/HSD1B ELISA Kit (LSBio, Seattle, WA, USA). Other details are listed in Supplemental materials.

### Animal preparation

The research protocol for experiment using animal was approved by the Animal Ethical Committee (Institutional Animal Care and Use Committee, IACUC, YWC-200217-1) of the Yonsei University Wonju College of Medicine, Wonju, Korea. All methods are reported in accordance with ARRIVE guidelines. Four-week-old *db/db* female mice (BKS.Cg-Dock7m+/+Leprdb/J) (n = 15), which were used as an animal model of DM, and C57BL/6J female mice (n = 15), which served as a control strain, were supplied by The Jackson Laboratory (Bar Harbor, ME, USA). The 11β-HSD1 inhibitor was dissolved in DMSO for topical application at a concentration of 100 µM^36^. DMSO was used as vehicle control. Vehicle and topical 11β-HSD1 inhibitors were applied twice a day to the dorsal surface of the mice for two weeks. All animals were housed in a standard environment with the temperature maintained at 22 ± 0.5 °C, relative humidity at 60 ± 5%, and a 12-h ⁄12-h light/dark cycle. After a one-week acclimatization period, the mice were fed a high-fat diet. In *db/db* mice, it is known that the degree of glucose intolerance develops with maturation; hyperglycemia usually occurs after 5 weeks, and the insulin concentration peaks at 3 months, with a maximum body weight of approximately 60 g. Accordingly, 8-week-old *db/db* mice were selected as the intermediate stage for the development of DM, and 14-week-old *db/db* mice were set as fully developed DM conditions.

Because we investigated the hormones involved in the HPA axis, we maintained a stress-minimized (handling-minimized) environment. Thus, one cage was used for each mouse. As *db/db* mice developed DM as they matured, the onset of symptoms due to polyuria and polyphagia became more frequent. Therefore, frequent changes in the cage and dietary supplementation were performed.

A constant day-night cycle and time of topical reagent application were maintained during the breeding period. Considering the circadian rhythm of mice, the experiment (collection of blood and specimen under anesthesia) was conducted between 1:00 and 3:00 PM, when the variation of cortisol was relatively lower compared to the early phase of the light-cycle^36,54,55^.

### Assessment of skin barrier function

Hair shaving was performed 3 days before the experiment under anesthesia (inhalant anesthesia with 2.5% sevoflurane) without interruption of the day-night cycle. TEWL was measured using a Tewameter (TM 300; Courage and Khazaka, Cologne, Germany), and SC hydration was measured using a Corneometer (CM 825; Courage and Khazaka). Basal TEWL was measured on the dorsal surface of the mice. Barrier recovery was determined by measuring TEWL immediately after and at 3 h after acute barrier disruption by tape stripping. The recovery rate was calculated as described previously. SC integrity was determined by measuring TEWL after four sequential strippings with D-squame® discs (CuDerm Corporation, Dallas, TX, USA)^13,15^.

### Quantification of SC cortisol

SC samples of the dorsal skin were collected from all mice by stripping off D-Squame® discs from their skin. The samples were placed in 500 μL of lysis buffer, vortexed, and incubated overnight at 4 °C. Cortisol levels in the collected protein extracts were measured using the corresponding ELISA kits (Aviva System Biology Corp., San Diego, CA, USA)^39^.

### Statistics

All data are expressed as mean ± standard error (SE). Statistical analyses were performed using unpaired Student’s *t*-tests and one-way ANOVA followed by the Bonferroni-Dunn test for multiple comparison.

## Supplementary Information

Supplementary Information 1.

Supplementary Information 2.

Supplementary Information 3.

Supplementary Information 4.

Supplementary Information 5.

## Data Availability

All data generated or analysed during this study are included in this article (and its supplementary files).
