# Supplementary material for: Hyperglycemia-activated 11β-hydroxysteroid dehydrogenase type 1 increases endoplasmic reticulum stress and skin barrier dysfunction
Source: Sci Rep. 2023 Jun 6;13:9206. doi: 10.1038/s41598-023-36294-y (PMC10244460; doi:10.1038/s41598-023-36294-y)
Supplement: Supplementary file 1 — Supplementary Information 1. [file 41598_2023_36294_MOESM1_ESM.docx]

**Hyperglycemia-activated 11β-hydroxysteroid dehydrogenase type 1 increases endoplasmic reticulum stress and skin barrier dysfunction**

Young Bin Lee^1^, Hyun Jee Hwang^1^, Eunjung Kim^1^, Sung Ha Lim^1^, Choon Hee Chung^2^,
Eung Ho Choi^1*^

*^1^Department of Dermatology, Yonsei University Wonju College of Medicine, Wonju, Korea,
^2^Department of Endocrinology and Metabolism, Yonsei University Wonju College of Medicine, Wonju, Korea.*

**SUPPLEMENTARY MATERIALS**

**Supplementary Figure 1.** In the MTT assay, a decrease in cell viability according to the concentration of the 11β-HSD1 inhibitor was definitive. To examine the efficacy of 11β-HSD1 inhibitor treatment on cell culture, it was necessary to select a concentration with little effect on cell viability.

**Supplementary Figure 2.** The MTT assay for 4-Phenyl butyric acid (4-PBA) showed that cell viability decreased in normal human keratinocyte culture as the concentration of 4-PBA increased.

**Supplementary Figure 3.** For the normoglycemic conditions, normal human keratinocytes were treated with thapsigargin (TG). A proportional increase in CHOP was observed as the concentration of TG increased. In addition, the 11β-HSD1 group showed an increased tendency after TG treatment compared to the controls.

**Supplementary Figure 4.** The *db/db* mice showed significantly higher weight and glucose concentrations than the control mice. **p*<0.05, compared to the value of 14-week-old *db/db* mice treated with the vehicle.

**Table S1. Primer sequences used in this study**

|  | Target | Forward primer (5’-3’) | Reverse primer (5’-3’) |
| --- | --- | --- | --- |
| C/EBPα | Human | TCAGCCGATATCAACACTTG' | AGTATCCGAGCAAAACCAAA |
| C/EBPβ | Human | GACAAGCACAGCGACGAGTA | AGCTGCTCCACCTTCTTCTG |
| CHOP | Human | AAGGCACTGAGCGTATCATGT | TGAAGATACACTTCCTTCTTG AACA |
| C/EBPα | Murine | TGGACAAGAACAGCAACGAGTAC | GCAGTTGCCCATGGCCTTGAC |
| C/EBPβ | Murine | TTCTACTACGAGCCCGACTGCC | CAGCTTGTCCACCGTCTTCTTG |
| CHOP | Murine | CCACCACACCTGAAAGCAGAA | AGGTGCCCCCAATTTCATCT |
| Filaggrin | Human | TGAAGCCTATGACACCACTGA | TCCCCTACGCTTTCTTGTCCT |
| Involucrin | Human | TCCTCCAGTCAATACCCATCAG | CAGCAGTCATGTGCTTTTCCT |
| Loricrin | Human | AGTGGACTGCGTGAAGAC | GCCAGAACCGCTGCTACC |

**Table S2. List of ELISA kits used in this cell culture study**

|  | Cat# | Vender |
| --- | --- | --- |
| CHOP | LS-F8872 | LSBio,  Seattle, WA, USA |
| RAGE | ab190807 | Abcam,  Boston, MA, USA |
| C/EBPα | LS-F32426 | LSBio,  Seattle, WA, USA |
| C/EBPβ | MBS2511196 | MyBioSource, San Diego, CA, USA |

ELISA, enzyme-linked immunosorbent assay

**Table S3. *p*-values of the *post hoc* analysis presented in Figure 2b (Cortisol level)**

|  | NG | HG | NG *si*Control | HG *si*Control |
| --- | --- | --- | --- | --- |
| NG | N/A | 0.018 | 0.092 | 0.001 |
| HG |  | N/A | 0.003 | <0.001 |
| NG *si*Control |  |  | N/A | 0.003 |
| HG *si*Control |  |  |  | N/A |

NG, Normoglycemic condition; HG, Hyperglycemic condition; *si*Control, scrambled siRNA control; N/A, not applicable

**Table S4. *p*-values of the *post hoc* analysis presented in Figure 4a (Fold change of 11β-HSD1)**

|  | NG (24h) | HG (24h) | 4-PBA (24h) | NG (48h) | HG (48h) | 4-PBA (48h) | NG (72h) | HG (72h) | 4-PBA (72h) |
| --- | --- | --- | --- | --- | --- | --- | --- | --- | --- |
| NG (24h) | N/A | 0.479 | 0.029 |  |  |  |  |  |  |
| HG (24h) |  | N/A | 0.062 |  |  |  |  |  |  |
| 4-PBA (24h) |  |  | N/A |  |  |  |  |  |  |
| NG (48h) |  |  |  | N/A | 0.022 | 0.991 |  |  |  |
| HG (48h) |  |  |  |  | N/A | 0.036 |  |  |  |
| 4-PBA (48h) |  |  |  |  |  | N/A |  |  |  |
| NG (72h) |  |  |  |  |  |  | N/A | 0.034 | 0.875 |
| HG (72h) |  |  |  |  |  |  |  | N/A | 0.049 |
| 4-PBA (72h) |  |  |  |  |  |  |  |  | N/A |

NG, Normoglycemic condition; HG, Hyperglycemic condition; 4-PBA, Hyperglycemic condition treated with 4-phenyl butyric acid; N/A, not applicable

**Table S5. *p*-values of the *post hoc* analysis presented in Figure 4a (Fold change of cortisol)**

|  | NG (24h) | HG (24h) | 4-PBA (24h) | NG (48h) | HG (48h) | 4-PBA (48h) | NG (72h) | HG (72h) | 4-PBA (72h) |
| --- | --- | --- | --- | --- | --- | --- | --- | --- | --- |
| NG (24h) | N/A | 0.048 | 0.019 |  |  |  |  |  |  |
| HG (24h) |  | N/A | 0.245 |  |  |  |  |  |  |
| 4-PBA (24h) |  |  | N/A |  |  |  |  |  |  |
| NG (48h) |  |  |  | N/A | 0.023 | 0.189 |  |  |  |
| HG (48h) |  |  |  |  | N/A | 0.089 |  |  |  |
| 4-PBA (48h) |  |  |  |  |  | N/A |  |  |  |
| NG (72h) |  |  |  |  |  |  | N/A | 0.002 | 0.003 |
| HG (72h) |  |  |  |  |  |  |  | N/A | 0.046 |
| 4-PBA (72h) |  |  |  |  |  |  |  |  | N/A |

NG, Normoglycemic condition; HG, Hyperglycemic condition; 4-PBA, Hyperglycemic condition treated with 4-phenyl butyric acid; N/A, not applicable

**RT-PCR of mRNA**
*Isolation of epidermis (murine experiment).* Skin samples were placed on the epidermis-side downward on Petri dishes, and subcutaneous fat was removed with a scalpel. Skin samples were then placed on the epidermis side up onto 10 mM EDTA in phosphate buffered saline and incubated at 37 °C for 30 min to separate the epidermis from the dermis. Following incubation in EDTA, the epidermis was scraped off with a scalpel, and total RNA was extracted.

*Total RNA preparation and cDNA synthesis*. Total RNA was extracted using a monophasic solution of phenol and guanidine isothiocyanate (TRIzol Reagent; Invitrogen, Carlsbad, CA, USA). RNA concentrations were determined using a UV spectrometer at 260 nm. Aliquots (1.0 μg) of RNA from each sample were reverse transcribed using the Moloney murine leukemia virus reverse transcriptase (MML-V RTase, Promega, Madison, WI, USA). Briefly, RNA samples were incubated at 80 °C for 5 min with molecular biology grade water. After incubation on ice, primer extension and reverse transcription were performed by adding 1× RT-buffer, 2 mM deoxynucleotide triphosphates (dNTPs, Promega, Madison, Wi, USA), 0.2 pM Oligo d(T) primer (Bioneer Inc., Korea), and MML-V RTase (2.5 units/μl) in 20 μl reaction volumes. Samples were then incubated at 42 °C for 60 min before storage at -20 °C.

*Quantitative PCR analysis of gene expression*. The expression of specific mRNAs was quantified using a Rotor-Gene 3000 (Corbett Life Science, Brisbane, Australia). Briefly, 10 μl PCR reactions were set up containing 1× Quantitect SYBR green PCR kit Master mix (Qiagen, Hilden, Germany), 8 mM manganese chloride, 200 μM dNTPs (Promega, Madison, WI, USA), 1.25 units of Hot start *Taq* polymerase, and 0.5 pM/μl primers. Approximately 60 ng of cDNA was used for each reaction. *GAPDH* was used as a housekeeping gene, enabling data to be expressed in relation to an internal reference to allow for differences in sampling. Data were obtained as Ct values (the cycle number at which logarithmic PCR plots cross a calculated threshold line) according to the manufacturer's guidelines, and these were used to determine ΔCt values (Ct of target gene − Ct of housekeeping gene) as raw data for gene expression. All reactions were performed in triplicate, and the results were expressed as the mean of values from three separate experiments. Samples were amplified using the primers outlined in Table S1 under the following conditions: 95 °C for 15 min, followed by 45 cycles of 95 °C for 15 s and 60 °C for 1 min.

**ELISA**

In a murine experiment, the serum level of AGE was determined using a mouse AGE ELISA kit (Uscn Life Science Inc., Wuhan, China), while the serum levels of ACTH and corticosterone were determined using a mouse ACTH ELISA kit (ALPCO Diagnostic, Salem, NH, USA) and a mouse corticosterone ELISA kit (Enzo Life Sci., Plymouth Meeting, PA, USA), respectively. The skin level of 11β-HSD1 was estimated using a mouse 11β-HSD1 kit (Wuhan Abebio Science Co., Wuhan, China). To detect serum levels of AGE, ACTH, and corticosterone, pre-dialysis blood samples were obtained after 12 h of overnight fasting. A small portion of the whole blood sample was centrifuged at 1000 × g for 15 min at room temperature. Serum samples were collected and frozen at -70 °C until ELISA. Protein samples for measuring the epidermal expression of 11β-HSD1 and murine epidermis were extracted using the Qproteome Mammalian Protein Prep kit (Qiagen, Hilden, Germany). The protein concentrations of the samples were determined using a Bradford assay, and equal amounts of protein were loaded onto each microplate well. Briefly, samples and standard solutions were added to plates pre-coated with an antibody and incubated at 37 °C for 2 h. Next, avidin conjugated to horseradish peroxidase was added to each microplate well and incubated. A TMB substrate solution was then added to each well. Only those wells that contain AGE, ACTH, corticosterone, 11β-HSD1, biotin-conjugated antibody, and enzyme-conjugated avidin will exhibit a change in color. The enzyme-substrate reaction was terminated by adding a sulfuric acid solution. The color change was measured using a microplate reader (BioTek Instruments, Inc., Winooski, VT, USA) at 450 ± 2 nm. The concentrations in the samples were determined by comparing the optical density of the samples to the standard curve. Measurements were performed in triplicate, and the results were averaged.
