# Supplementary figures and images for: Hyperglycemia-activated 11β-hydroxysteroid dehydrogenase type 1 increases endoplasmic reticulum stress and skin barrier dysfunction
Source: Sci Rep. 2023 Jun 6;13:9206. doi: 10.1038/s41598-023-36294-y (PMC10244460; doi:10.1038/s41598-023-36294-y)

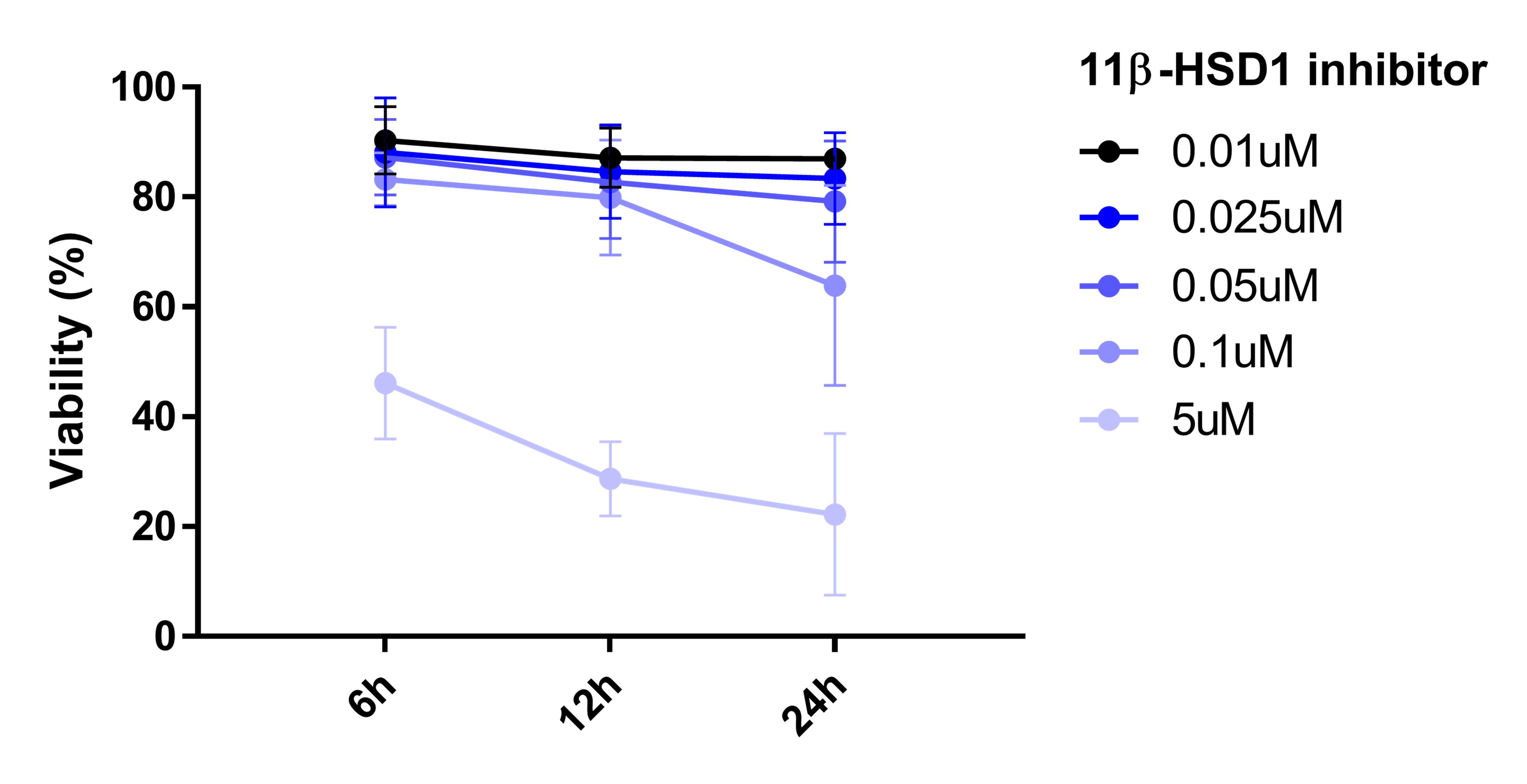

Supplement: Supplementary file 2 — Supplementary Information 2. [file 41598_2023_36294_MOESM2_ESM.jpg]

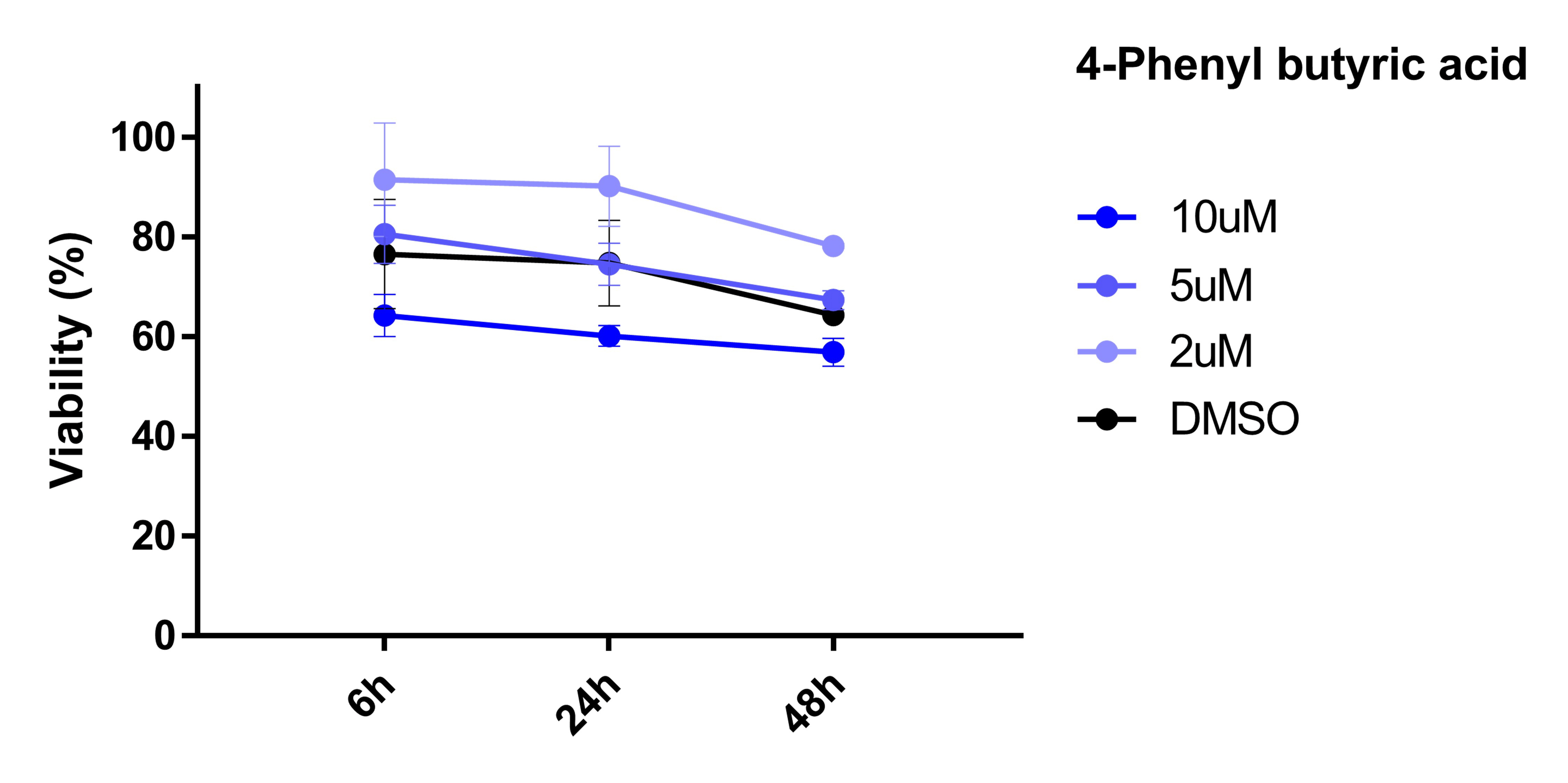

Supplement: Supplementary file 3 — Supplementary Information 3. [file 41598_2023_36294_MOESM3_ESM.jpg]

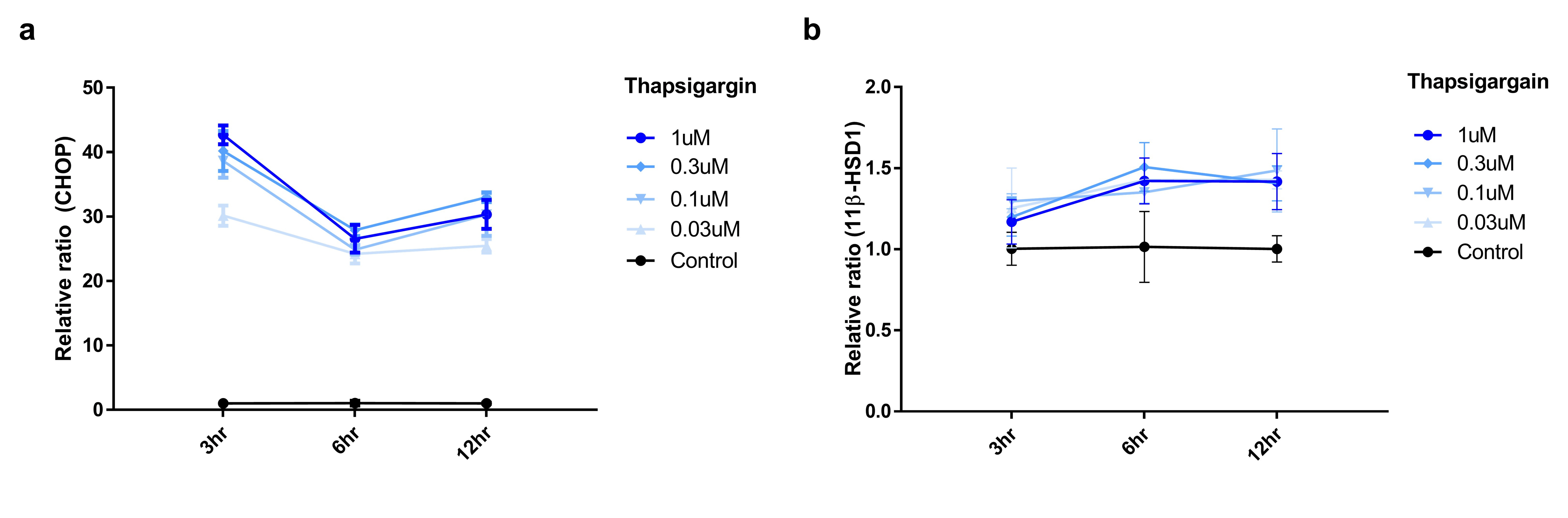

Supplement: Supplementary file 4 — Supplementary Information 4. [file 41598_2023_36294_MOESM4_ESM.jpg]

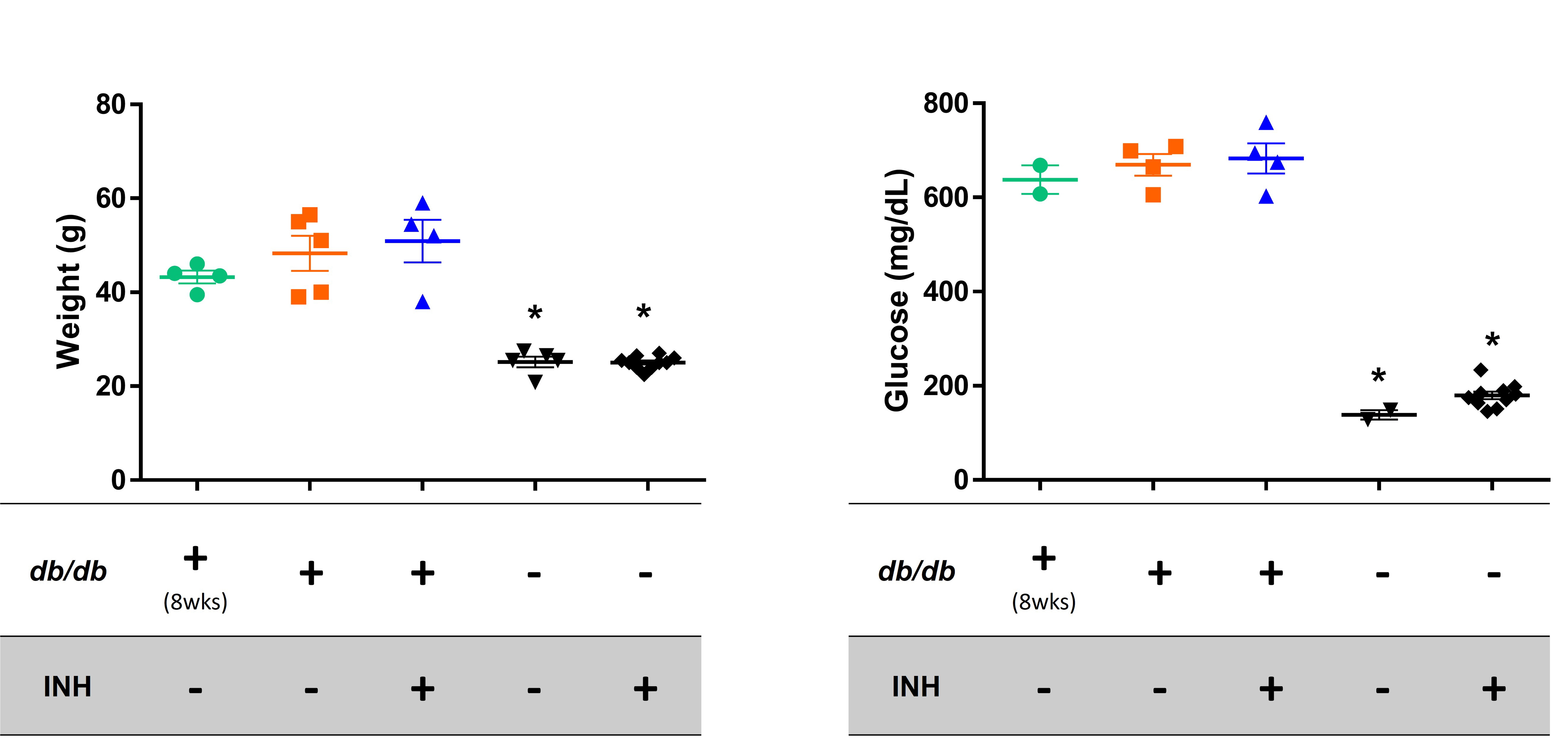

Supplement: Supplementary file 5 — Supplementary Information 5. [file 41598_2023_36294_MOESM5_ESM.jpg]
